# Supplementary material for: Efficient circular RNA synthesis for potent rolling circle translation
Source: Nat Biomed Eng. 2024 Dec 13;9(7):1062–74. doi: 10.1038/s41551-024-01306-3 (PMC12270912; doi:10.1038/s41551-024-01306-3)
Supplement: Supplementary file 1 — Reporting Summary [file 41551_2024_1306_MOESM1_ESM.pdf]

nature portfolio

Corresponding author(s): Yifei Du, Venki Ramakrishnan

Last updated by author(s): Sep 5, 2024

Reporting Summary

Nature Portfolio wishes to improve the reproducibility of the work that we publish. This form provides structure for consistency and transparency in reporting. For further information on Nature Portfolio policies, see our Editorial Policies and the Editorial Policy Checklist.

Statistics

For all statistical analyses, confirm that the following items are present in the figure legend, table legend, main text, or Methods section.

n/a	Confirmed	
☐	☒	The exact sample size (n) for each experimental group/condition, given as a discrete number and unit of measurement
☐	☒	A statement on whether measurements were taken from distinct samples or whether the same sample was measured repeatedly
☐	☒	The statistical test(s) used AND whether they are one- or two-sided<br>*Only common tests should be described solely by name; describe more complex techniques in the Methods section.*
☒	☐	A description of all covariates tested
☐	☒	A description of any assumptions or corrections, such as tests of normality and adjustment for multiple comparisons
☐	☒	A full description of the statistical parameters including central tendency (e.g. means) or other basic estimates (e.g. regression coefficient) AND variation (e.g. standard deviation) or associated estimates of uncertainty (e.g. confidence intervals)
☐	☒	For null hypothesis testing, the test statistic (e.g. F, t, r) with confidence intervals, effect sizes, degrees of freedom and P value noted<br>*Give P values as exact values whenever suitable.*
☒	☐	For Bayesian analysis, information on the choice of priors and Markov chain Monte Carlo settings
☒	☐	For hierarchical and complex designs, identification of the appropriate level for tests and full reporting of outcomes
☒	☐	Estimates of effect sizes (e.g. Cohen's d, Pearson's r), indicating how they were calculated

Our web collection on statistics for biologists contains articles on many of the points above.

Software and code

Policy information about availability of computer code

Data collection	No code was generated in this research. Software used for data collection are: ViiA 7 Real-Time PCR System, Quantstudio Real-time PCR software v1.3, Bio-Rad ChemiDoc XRS+ Imaging System, EVOS FL microscope, Spark 10M plate reader.
Data analysis	Software used for data analysis are: Microsoft Excel, Image J

For manuscripts utilizing custom algorithms or software that are central to the research but not yet described in published literature, software must be made available to editors and reviewers. We strongly encourage code deposition in a community repository (e.g. GitHub). See the Nature Portfolio guidelines for submitting code & software for further information.

Data

Policy information about availability of data

All manuscripts must include a data availability statement. This statement should provide the following information, where applicable:

- Accession codes, unique identifiers, or web links for publicly available datasets
- A description of any restrictions on data availability
- For clinical datasets or third party data, please ensure that the statement adheres to our policy

All sequences are provided as Supplementary information. Source data for the figures are provided with this paper. The raw and analysed datasets generated during the study are available for research purposes from the corresponding authors on reasonable request.

Research involving human participants, their data, or biological material

Policy information about studies with human participants or human data. See also policy information about sex, gender (identity/presentation), and sexual orientation and race, ethnicity and racism.

Reporting on sex and gender	The study did not involve human research participants.
Reporting on race, ethnicity, or other socially relevant groupings	–
Population characteristics	–
Recruitment	–
Ethics oversight	–

Note that full information on the approval of the study protocol must also be provided in the manuscript.

Field-specific reporting

Please select the one below that is the best fit for your research. If you are not sure, read the appropriate sections before making your selection.

☒ Life sciences ☐ Behavioural & social sciences ☐ Ecological, evolutionary & environmental sciences

For a reference copy of the document with all sections, see nature.com/documents/nr-reporting-summary-flat.pdf

Life sciences study design

All studies must disclose on these points even when the disclosure is negative.

Sample size	3-4 biological or independent replicates were used in all experiments to allow for statistical analysis.
Data exclusions	For the RT-qPCR experiments, 3 replicates were used for each biological sample. When the difference between the CT of a replicate and the CT of the remainder two replicates was larger than 1, this replicate was removed. Otherwise, no data were excluded from analysis.
Replication	All findings were replicated at least twice, once in the presented experiments and at least once in preliminary tests.
Randomization	Randomization was not used.
Blinding	Blinding was not needed.

Reporting for specific materials, systems and methods

We require information from authors about some types of materials, experimental systems and methods used in many studies. Here, indicate whether each material, system or method listed is relevant to your study. If you are not sure if a list item applies to your research, read the appropriate section before selecting a response.

Materials & experimental systems

n/a	Involved in the study	
☐	☒	Antibodies
☐	☒	Eukaryotic cell lines
☒	☐	Palaeontology and archaeology
☒	☐	Animals and other organisms
☒	☐	Clinical data
☒	☐	Dual use research of concern
☒	☐	Plants

Methods

n/a	Involved in the study	
☒	☐	ChIP-seq
☒	☐	Flow cytometry
☒	☐	MRI-based neuroimaging

Antibodies

Antibodies used	Monoclonal ANTI-FLAG® M2 antibody produced in mouse (F1804, Sigma-Aldrich); Goat anti-Mouse IgG (H+L) Highly Cross-Adsorbed Secondary Antibody, Alexa Fluor™ Plus 800 (A32730, Invitrogen); HRP Mouse monoclonal [mAbcam 8226] to beta Actin (ab20272, abcam).

Validation

The ANTI-FLAG M2 mouse, affinity purified monoclonal antibody binds to fusion proteins containing a FLAG peptide sequence.The antibody recognizes the FLAG peptide sequence at the N-terminus, Met-N-terminus,C-terminus, and internal sites of the fusion protein. The manufacturer shows a immunofluorescence result that indicates the validation of the product.
https://www.sigmaaldrich.com/GB/en/product/sigma/f1804?utm_source=google&utm_medium=cpc&utm_campaign=15001183107&utm_content=127306761543&gclid=EAIaIQobChMIyrOr-JC7hQMVT5RQBh1CoAU6EAAYASAAEgIrcvD_BwE
HRP Mouse monoclonal [mAbcam 8226] to beta Actin, widely used as loading control, the manufacturer shows three western blot results that indicated the validation of the product. https://www.abcam.com/products/primary-antibodies/hrp-beta-actin-antibody-mabcam-8226-loading-control-ab20272.html

Eukaryotic cell lines

Policy information about cell lines and Sex and Gender in Research

Cell line source(s)

HEK 293F cells were from Thermo Fisher, A549 cells were from Dr. Alba Herrero Del Valle.

Authentication

The cell lines were not authenticated.

Mycoplasma contamination

The cell lines were not tested for mycoplasma contamination.

Commonly misidentified lines
(See ICLAC register)

No commonly misidentified cell lines were used.
